# Electrochemical Nitrogen Reduction: The Energetic Distance to Lithium

**DOI:** 10.1021/acsenergylett.4c01638

**Published:** 2024-09-19

**Authors:** Alexander Bagger, Romain Tort, Maria-Magdalena Titirici, Aron Walsh, Ifan E. L. Stephens

**Affiliations:** † Department of Physics, Technical University of Denmark, Kongens Lyngby 2800, Denmark; ‡ Department of Chemical Engineering, Imperial College London, SW7 2AZ London, United Kingdom; ¶ Department of Materials, Imperial College London, London SW7 2AZ, United Kingdom

## Abstract

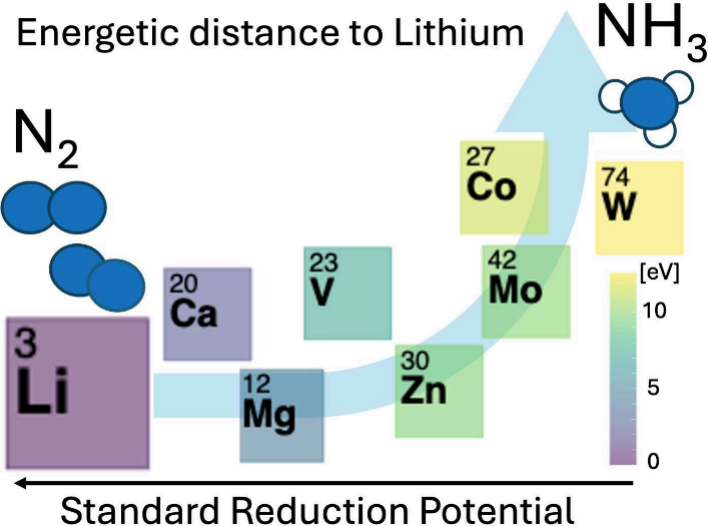

Energy-efficient electrochemical reduction of nitrogen to ammonia could help in mitigating climate change. Today, only Li- and recently Ca-mediated systems can perform the reaction. These materials have a large intrinsic energy loss due to the need to electroplate the metal. In this work, we present a series of calculated energetics, formation energies, and binding energies as fundamental features to calculate the energetic distance between Li and Ca and potential new electrochemical nitrogen reduction systems. The featured energetic distance increases with the standard potential. However, dimensionality reduction using principal component analysis provides an encouraging picture; Li and Ca are not exceptional in this feature space, and other materials should be able to carry out the reaction. However, it becomes more challenging the more positive the plating potential is.

The reduction of N_2_ to NH_3_ is a critical process for the growth of plants in nature and food in crops, for the chemical industry, and as an energy carrier. Although dinitrogen (N_2_) is highly abundant in the atmosphere, it does not react easily and is extremely difficult to activate.

At high temperatures and pressures activating N_2_ is possible via the industrial Haber–Bosch process^1^ with high energy efficiency thanks to a highly integrated process and economy of scale. However, it consumes H_2_ typically delivered from steam methane reforming (SMR), resulting in immense energy consumption and CO_2_ emissions. This limitation has driven researchers toward the discovery of alternatives.

At ambient conditions, different routes prevail including enzymatic, homogeneous, and electrocatalytic activation.^2^ The enzymatic activation of N_2_ happens in nature by the nitrogenase enzyme. The active site in the enzyme appears to be iron as an essential transition metal, and it typically contains molybdenum (FeMo-nitrogenase is the most common form),^3,4^ with a middle carbon atom.^5^ The operation of nitrogenase at ambient conditions to allow nitrogen reduction to ammonia has been long debated,^6^ and new findings for nitrogenase are still relevant today.^7,8^ Homogeneous activation is inspired by the observation of the nitrogenase enzyme and is driven by well-defined molecular coordination complexes.^9^ In the first instance, the complexes only fixated nitrogen.^10^ Later they facilitated protonation of nitrogen,^11^ and finally conversion to ammonia was achieved.^12−14^

Electrocatalytic activation and direct electrochemical N_2_ reduction to NH_3_ could provide a sustainable alternative for small-scale production. The only electrode upon which multiple groups have provided unequivocal evidence that electrochemical nitrogen reduction can take place is in situ deposited Li in an organic electrolyte.^15−19^ The discovery of a working Ca system was recently provided by Fu et al.,^20^ while a two-step electrochemical ammonia synthesis has been shown on Mg,^21^ proving Mg to form a metal-nitride and subsequently dissolve it to yield ammonia. However, no continuous production with Mg has been shown to date. Other systems such as metal electrodes in aqueous solution produce such low yields of ammonia that it is impossible to distinguish it from background contamination.^22^ In optimizing the Li-based system, researchers have been successful in achieving close to 100% selectivity for nitrogen reduction to ammonia.^18^ Although the Li (and Ca) based route shows a viable pathway to ammonia, both systems suffer from a low energy efficiency of ∼28% due to the −3 V plating potential. A recent analysis compares the energy efficiency of these electrochemical systems with the Haber–Bosch process,^23^ suggesting a maximum cell potential of 0.38 V to reach energy parity with Haber–Bosch and ruling out alkali metals such as Li and Ca on that single metric. Thus, these systems can only compete when ammonia price is not the key metric and rather soft parameters dominate, such as handling and production on-site, and for small/limited/specialized usage.

There is a pressing need to establish the features that enable electrochemical nitrogen reduction under ambient conditions.^24^ Using density functional theory (DFT), we previously searched for the rules of nitrogen fixation.^25^ Several metals, including Mg, Ca, Cr, and Mo, showed a similar reactivity to Li toward N_2_, both in terms of adsorption energy and the propensity to form a bulk nitride. In a separate study,^20^ Fu et al. discovered that Ca can also electrochemically convert N_2_ to NH_3_, giving credence to our predictions (notably, our experiments were unsuccessful). It would seem that the electrochemical route is dependent on a unique combination of properties that may not only be catalytic. Properties that have been suggested are dinitrogen binding,^7^ nitrogen binding,^26^ nitrogen dissociation,^27^ and the transport of reactants and products through the solid–electrolyte interphase layer (SEI).^28^ In particular, the functionality of the SEI layer is difficult to probe in experiments and computations. In the battery literature, one can find insights into the SEI in Al, Mg^29,30^, and Ca^31−33^ batteries. However, the SEI for a battery needs to be ion conductive but otherwise passive, while the SEI for a nitrogen reduction system has the requirement that reactants and proton sources can reach the material and products can leave the material through the SEI.^34^ Thus, insights can be found in battery literature but are not necessarily translatable to electrochemical nitrogen reduction.

In this work, we investigate the energetic distances of materials from Li and Ca as electrodes capable of electrochemical conversion of N_2_ to NH_3_. We focus on calculating a series of material properties across the periodic table from first principles: phase formation energies (Δ*H*_*name*_), reaction energies between phases (Δ*E*_*M*_*x*__), and binding energies on surfaces (ΔE_*N_), where the subscripts *name*, *M*_*x*_, and *N refer to the name of formation energies, name of phase reaction (*M* being the element in the periodic table), and binding energy, respectively. These calculated phase energies and binding energies provide a solid data set to capture trends across the periodic table and allow us to estimate energetic distances to Li and Ca. We then hypothesize that the most likely materials to work for electrochemical nitrogen reduction are materials with the shortest distances to Li and Ca. All data is provided in Tables S1–S3, and examples of calculated DFT structures of Li are given in Figure S1.

Examples of reactions for formation energies (Δ*H*_*Nitride*_), reaction energies between phases (Δ*E*_*M*_*x*__), and binding energies on surfaces (ΔE_*N_) are
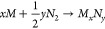
1
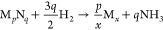
2
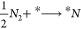
3From the reactions, we calculate these properties as
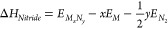
4
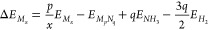
5
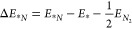
6Here the asterisks, *, refers to a reaction intermediate on the electrode surface, E is the obtained DFT energy, and the rest of the equations are given in the Supporting Information. In total this allows us to investigate 16 different materials features; 8 different phase formation energies (Δ*H*_*Nitride*_, Δ*H*_*Hydride*_, Δ*H*_*Oxide*_, Δ*H*_*M*_*x*_*O*_*y*_*H*_*z*__, Δ*H*_*M*_*x*_*C*_*y*_*O*_*z*__, Δ*H*_*M*_*x*_*F*_*y*__, Δ*H*_*XN*_3__, Δ*H*_*X*_3_*N*_), and 4 phase reaction energies (Δ*E*_*M*_*x*_*O*_*y*_*H*_*z*__, Δ*E*_*M*_*x*_*O*_*y*__, Δ*E*_*M*_*x*_*H*_*y*__, Δ*E*_*M*_*x*__), and 4 binding energies (ΔE_*N_, ΔE_*N_2__, ΔE_*NH_2__, ΔE_*NH_3__).

For some elements of the periodic table, certain features (such as Δ*H*_*M*_*x*_*C*_*y*_*O*_*z*__) would not have a corresponding defined structure or would cause difficulties in converging DFT simulations. In such cases, we carried out linear regressions to predict missing values, as shown in Figure S5, and values are colored red in Tables S1–S3.

The crystalline phases are selected as the most stable structure from the Materials Project database.^35^ If the most stable nitride phase has isolated nitrogen atoms we denoted it the “cleaved phase”, and if it does not have isolated nitrogen atoms we denote it the “coupling phase”, similar to our previous work.^25^ To assess the formation of the correct SEI layer we have calculated the stability of bulk ionic salt phases, specifically Δ*H*_*M*_*x*_*O*_*y*_*H*_*z*__,^36,37^ Δ*H*_*M*_*x*_*C*_*y*_*O*_*z*__,^38,39^ and Δ*H*_*M*_*x*_*F*_*y*__^18,40^ phases. We chose these compounds as descriptors toward the formation of organic or inorganic SEI species as our earlier study suggested that electrochemical nitrogen reduction is facilitated by the presence of an inorganic SEI.^36^ For binding energies, we use only nitrogen reduction reaction intermediates, but several additional features could, in principle, be included in an extended analysis.

For the above-discussed materials properties, we use statistical analysis to

- Display the *Z*-score for Li and Ca for each material property. The score is given as, *Z* = (*x* – μ)/σ, where *x* is the value of Li or Ca, μ is the mean, and σ is the standard deviation obtained from the material property. Values of *Z* above 3 (or ∼2) indicate that the material is an outlier or exceptional material with respect to the entire data set.
- Calculate the energetic distance to Li and Ca. This will show which material across the periodic table has overall similar thermodynamic characteristics to Li and Ca.
- Use principal component analysis (PCA) of scaled features to project the feature space down to two dimensions and assess which materials are closest to Li and Ca.

The binding energies of nitrogen (ΔE_*N_) and the calculated energetic distance to Li versus the standard reduction potential are shown for the materials across the periodic table in Figure 1. The atomic nitrogen binding energy of Li and Ca is neither weak nor strong, which could correspond to a Sabatier principle (Figure 1a). However, multiple other elements have close to similar binding (Sr, Ba, Nb, Fe, W, Mo, etc.) which suggests that on the basis of the atomic nitrogen alone these could electrochemically reduce N_2_ to NH_3_, as we previously suggested.^25^ The notion that other materials should function is also supported by the statistical standard scores provided in the plot for Li and Ca, which do not show any statistical significance as the score is well below 2. The standard score *Z*_Li_ = −0.34 and *Z*_Ca_ = −0.91 for ΔE_*N_ means that both materials have a ΔE_*N_ value within 0.34 and 0.91 standard deviation of the mean for this material property, respectively. Analysis of all additional features versus the standard reduction potential is shown in Figure S2. We do not, in any case, find significant values for both Li and Ca, showing that none of these materials are exceptional or outliers with respect to singular material features used in this work. The most significant values are Z = −2.13 for Li with the formation energy of Li_3_N phase, while *Z* = −2.17 for Ca with the binding energy of *N_2_. It should also be noted that in particular, the binding energy of nitrogen (ΔE_*N_) varies quite a lot for s-block elements depending on the lattice constant of the unit cell (see convergence checks in Figure S3 and S4).

**Figure 1:**
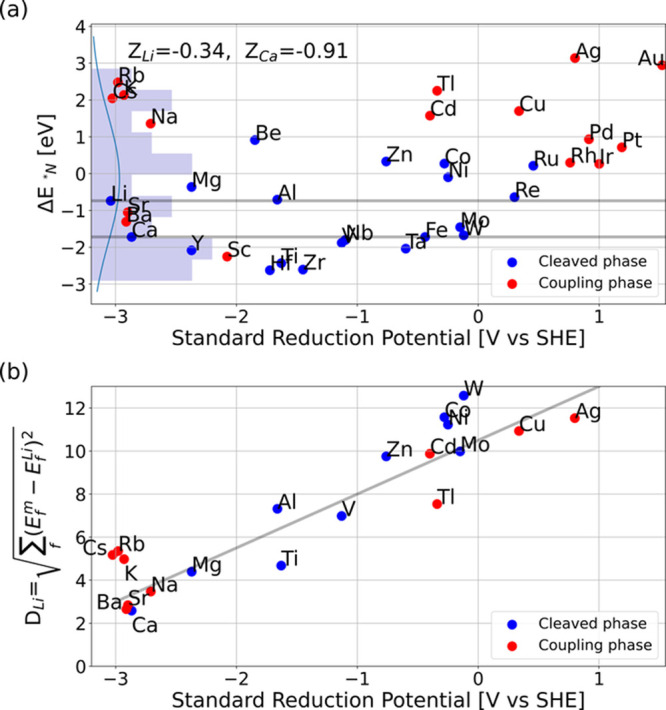
(a) Calculated binding energies of nitrogen (ΔE_*N_) versus standard reduction potential. Horizontal lines indicate Li and Ca (working electrodes). A histogram and a probability density distribution are plotted together with the Z-score values. Neither Li nor Ca is exceptional in that regard, as a Z-score >2 gives a data point outside of 95% of the data assuming a normal distribution. (b) Calculated distance to Li, with *m* being the metals and *f* the features, such as the formation energies and binding energies, plotted as a function of the standard reduction potential. Ca is the material closest to Li, and with increasing standard potential the further away the materials energetics are. “Cleaved phase” means that the material forms a nitride phase with isolated nitrogen atoms.

To compare all features in one dimension versus standard reduction potential, we define an effective energetic distance from the formation energies and binding energies as
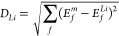
7with *m* being the metals and *f* the features. Interestingly, this analysis shown in Figure 1b depicts that the closest energetically material to Li is Ca. The performance of Ca has only recently been demonstrated.^20^ This highlights the fact that the energetic distance calculations could provide opportunities for close-lying materials. However, it also displays that materials which have close to similar nitrogen binding (ΔE_*N_) to Li, such as W, Mo, etc. as shown in Figure 1a, can have a distance very far from Li when including multiple material features. Unfortunately, it can even be observed that the material’s distance to Li as a function of plating potential is almost linear, depicting that material similar to Li will have similar energy efficiency. This also implies that an energy-efficient electrochemical system will use a different chemistry, hence ruling out battery chemistries in the future. To display the importance of having less plating potential, a recent energy efficiency analysis has been provided.^23,41^

While the scalar distance to Li provides an effective visualization, it may be hiding more complex patterns, and some features with high variation could carry the weight of the distance. It can be helpful to scale all features to obtain similar weights and carry out dimensionality reduction. We choose PCA to reduce the dimensions while preserving as much of the variance in the data as possible, and previously PCA has been successful in obtaining insights into the CO_2_ reduction reaction, which also depends on a complex multifeature space.^42^ In the PCA the data is linearly transformed into a new coordinate system such that the directions (principal components) capture the largest variation in the data. This means that principal component 1 captures the most of the variation, principal component 2 the second most, etc., until the number of principal components hits the original data set dimensions and the explained variance reaches 100%. We have exemplified this in Figure S7.

A two-dimensional PCA was applied for (a) DFT calculated energies only and (b) the inclusion of predicted values where DFT values were missing (see red data in Tables S1–S3, and analysis in Figure S5), and the results are provided in Figure 2. For both plots, around ∼67% and ∼14% of the variation in the data set is captured by the first and second principal components, respectively. This gives a total of ∼81% explained variations by only these two dimensions (see also Figure S7). We have colored Li and Ca with a larger blue point to show these working materials at the perimeter in the plots but with several nearby materials showing again that neither Li nor Ca are unique. We have also co-plotted the original feature dimensions with the green length as weight in the plot to display their direction projected into the first and second principal components, as this can help to interpret the meaning of the principal component. Note that some features point in a similar direction, which means that these features are more correlated than others (e.g., ΔE_*NH_3__ and Δ*H*_Nitride_) in Figure 2a.

**Figure 2:**
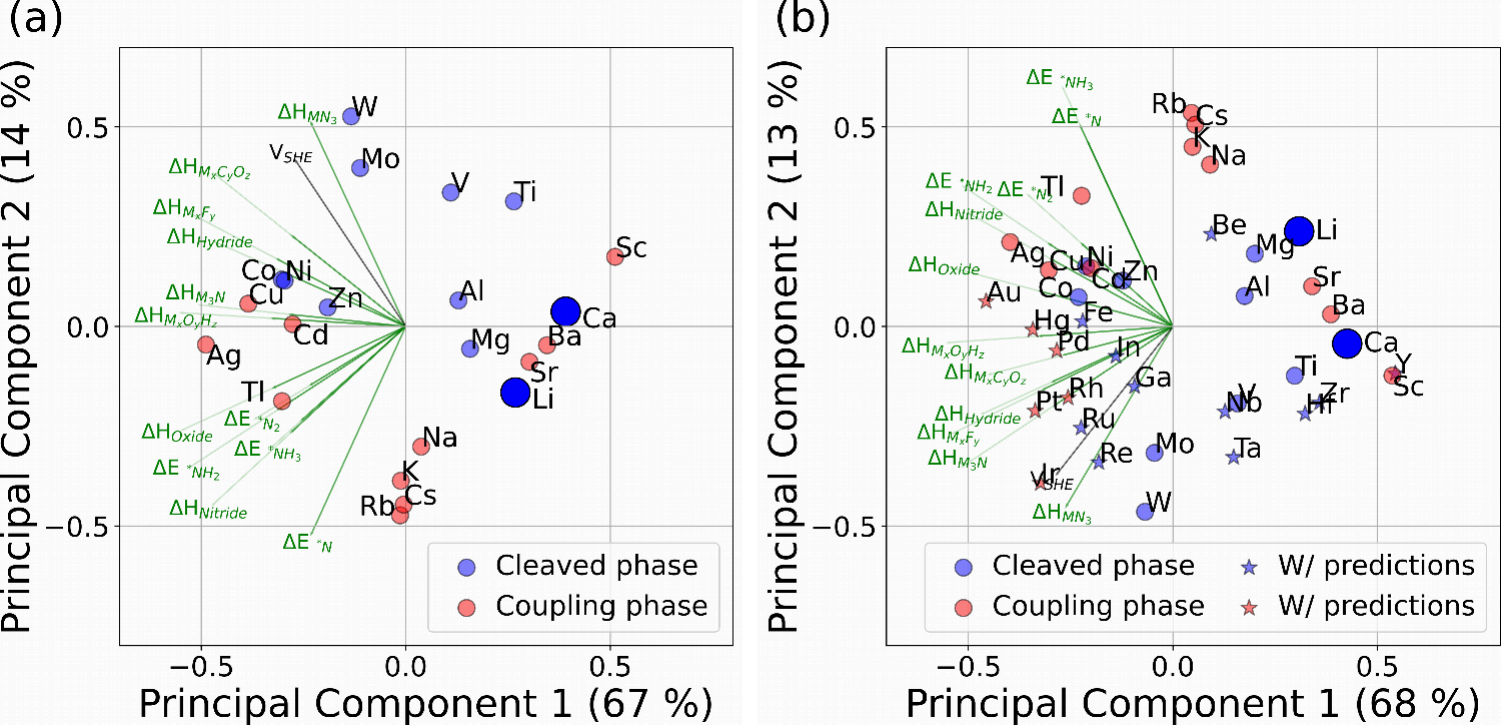
Principal component analysis of the nitrogen reduction feature space: formation energies (Δ*H*), binding energies (Δ*E*), and the standard reduction potential (*V*_SHE_) for (a) DFT energies and (b) including data with linear regression predicted values noted by star points. “Cleave phase” means that the material forms a nitride phase with isolated nitrogen atoms. Data are shown in Tables S1 and S2.

Among materials that have a nitride phase with isolated nitrogen atoms (denoted “cleaved phase”), Mg and Al are close to Li (Figure 2a). We have recently tested these materials with selected salt and electrolyte components.^25^ Using the predicted values in Figure 2b shows that also Ti, Be, Zr, or Hf look interesting. However, it is not given that any of these materials form an SEI layer suitable for sustained electrocatalysis, and ideally an electrolyte interphase should be formed in the aqueous electrolyte as investigated for aqueous batteries.^43^

Since reduction potential is a property we would like to constrain to positive values, we performed a similar PCA (Figure S8), excluding this parameter from our variables. This clearly shows that there is a negligible change in PCAs. One can also see in Figure 2a,b that the materials of interest lie almost on a line orthogonal to the co-plotted original *V*_SHE_ component, which challenges energy-efficient improvements.

In conclusion, our analysis depicts that Mg or Al is most likely to work, given that they are battery materials (can form a stable SEI), have a close distance to both Li and Ca, and form a dissociative nitride phase. If a dissociative nitride phase is not needed, one should also experiment with Sr, Ba, and Sc. Unfortunately, all of these single-component materials would imply a marginal improvement in the energy efficiency of the system. To bring substantial improvement one could search for complex multicomponent electrodes or a completely different setup such as intermediate temperature electrocatalysis (improving kinetics), high nitrogen pressure (improving nitrogen coverage), or a controllable artificial transport layer (moderate access to protons and metal cations but facilitate access to N_2_).

## Supporting Information Available

Computational details, calculations of features, convergence checks, data in table format and filling of missing data, and additional plots (PDF)

## References

[ref1] Ertl G. Primary steps in catalytic synthesis of ammonia. Journal of Vacuum Science & Technology A 1983, 1, 1247–1253. 10.1116/1.572299.

[ref2] Westhead O.; Barrio J.; Bagger A.; Murray J. W.; Rossmeisl J.; Titirici M. M.; Jervis R.; Fantuzzi A.; Ashley A.; Stephens I. E. Near ambient N_2_ fixation on solid electrodes versus enzymes and homogeneous catalysts. Nature Reviews Chemistry 2023, 7, 184–201. 10.1038/s41570-023-00462-5. 37117902

[ref3] Burgess B. K.; Lowe D. J. Mechanism of Molybdenum Nitrogenase. Chem. Rev. 1996, 96, 2983–3012. 10.1021/cr950055x. 11848849

[ref4] Eady R. R. Structure-Function Relationships of Alternative Nitrogenases. Chem. Rev. 1996, 96, 3013–3030. 10.1021/cr950057h. 11848850

[ref5] Einsle O.; Tezcan F. A.; Andrade S. L. A.; Schmid B.; Yoshida M.; Howard J. B.; Rees D. C. Nitrogenase MoFe-Protein at 1.16 Å Resolution: A Central Ligand in the FeMo-Cofactor. Science 2002, 297, 1696–1700. 10.1126/science.1073877. 12215645

[ref6] Chatt J.; Dilworth J. R.; Richards R. L. Recent advances in the chemistry of nitrogen fixation. Chem. Rev. 1978, 78, 589–625. 10.1021/cr60316a001.

[ref7] Bagger A.; Wan H.; Stephens I. E. L.; Rossmeisl J. Role of Catalyst in Controlling N_2_ Reduction Selectivity: A Unified View of Nitrogenase and Solid Electrodes. ACS Catal. 2021, 11, 6596–6601. 10.1021/acscatal.1c01128.

[ref8] Bukas V. J.; Norskov J. K. A Molecular-Level Mechanism of the Biological N_2_ Fixation. ChemRxiv 2019, 1 10.26434/chemrxiv.10029224.v1.

[ref9] Masero F.; Perrin M. A.; Dey S.; Mougel V. Dinitrogen Fixation: Rationalizing Strategies Utilizing Molecular Complexes. Chemistry–A European Journal 2021, 27, 3892–3928. 10.1002/chem.202003134. 32914919 PMC7986120

[ref10] Allen A. D.; Senoff C. V. Nitrogenopentammineruthenium(II) complexes. Chem. Commun. (London) 1965, 24, 621–622. 10.1039/c19650000621.

[ref11] Chatt J.; Heath G. A.; Richards R. L. The reduction of ligating dinitrogen to yield a ligating N_2_H_2_ moiety. J. Chem. Soc., Chem. Commun. 1972, 18, 1010–1011. 10.1039/c39720001010.

[ref12] Yandulov D. V.; Schrock R. R. Catalytic Reduction of Dinitrogen to Ammonia at a Single Molybdenum Center. Science 2003, 301, 76–78. 10.1126/science.1085326. 12843387

[ref13] Arashiba K.; Miyake Y.; Nishibayashi Y. A molybdenum complex bearing PNP-type pincer ligands leads to the catalytic reduction of dinitrogen into ammonia. Nat. Chem. 2011, 3, 120–125. 10.1038/nchem.906. 21258384

[ref14] Anderson J. S.; Rittle J.; Peters J. C. Catalytic conversion of nitrogen to ammonia by an iron model complex. Nature 2013, 501, 84–87. 10.1038/nature12435. 24005414 PMC3882122

[ref15] Andersen S. Z.; Čolić V.; Yang S.; Schwalbe J. A.; Nielander A. C.; McEnaney J. M.; Enemark-Rasmussen K.; Baker J. G.; Singh A. R.; Rohr B. A.; Statt M. J.; Blair S. J.; Mezzavilla S.; Kibsgaard J.; Vesborg P. C. K.; Cargnello M.; Bent S. F.; Jaramillo T. F.; Stephens I. E. L.; No̷rskov J. K.; Chorkendorff I. A rigorous electrochemical ammonia synthesis protocol with quantitative isotope measurements. Nature 2019, 570, 504–508. 10.1038/s41586-019-1260-x. 31117118

[ref16] Tsuneto A.; Kudo A.; Sakata T. Lithium-mediated electrochemical reduction of high pressure N2 to NH3. J. Electroanal. Chem. 1994, 367, 183–188. 10.1016/0022-0728(93)03025-K.

[ref17] Lazouski N.; Schiffer Z. J.; Williams K.; Manthiram K. Understanding Continuous Lithium-Mediated Electrochemical Nitrogen Reduction. Joule 2019, 3, 1127–1139. 10.1016/j.joule.2019.02.003.

[ref18] Du H. L.; Chatti M.; Hodgetts R. Y.; Cherepanov P. V.; Nguyen C. K.; Matuszek K.; MacFarlane D. R.; Simonov A. N. Electroreduction of nitrogen with almost 100% current-to-ammonia efficiency. Nature 2022, 609, 722–727. 10.1038/s41586-022-05108-y. 35868345

[ref19] Izelaar B.; Ripepi D.; van Noordenne D. D.; Jungbacker P.; Kortlever R.; Mulder F. M. Identification, Quantification, and Elimination of NO_x_ and NH_3_ Impurities for Aqueous and Li-Mediated Nitrogen Reduction Experiments. ACS Energy Letters 2023, 8, 3614–3620. 10.1021/acsenergylett.3c01130. 37588017 PMC10425974

[ref20] Fu X.; Niemann V. A.; Zhou Y.; Li S.; Zhang K.; Pedersen J. B.; Saccoccio M.; Andersen S. Z.; Enemark-Rasmussen K.; Benedek P.; Xu A.; Deissler N. H.; Mygind J. B. V.; Nielander A. C.; Kibsgaard J.; Vesborg P. C. K.; No̷rskov J. K.; Jaramillo T. F.; Chorkendorff I. Calcium-mediated nitrogen reduction for electrochemical ammonia synthesis. Nat. Mater. 2024, 23, 101–107. 10.1038/s41563-023-01702-1. 37884670

[ref21] Krebsz M.; Hodgetts R. Y.; Johnston S.; Nguyen C. K.; Hora Y.; MacFarlane D. R.; Simonov A. N. Reduction of dinitrogen to ammonium through a magnesium-based electrochemical process at close-to-ambient temperature. Energy Environ. Sci. 2024, 17, 4481–4487. 10.1039/D4EE01090F.

[ref22] Choi J.; Suryanto B. H. R.; Wang D.; Du H.-L.; Hodgetts R. Y.; Ferrero Vallana F. M.; MacFarlane D. R.; Simonov A. N. Identification and elimination of false positives in electrochemical nitrogen reduction studies. Nat. Commun. 2020, 11, 5546 10.1038/s41467-020-19130-z. 33144566 PMC7641139

[ref23] Jin D.; Chen A.; Lin B.-L. What Metals Should Be Used to Mediate Electrosynthesis of Ammonia from Nitrogen and Hydrogen from a Thermodynamic Standpoint?. J. Am. Chem. Soc. 2024, 146, 12320–12323. 10.1021/jacs.4c02754. 38597430

[ref24] Westhead O.; Jervis R.; Stephens I. E. L. Is lithium the key for nitrogen electroreduction?. Science 2021, 372, 1149–1150. 10.1126/science.abi8329. 34112680

[ref25] Tort R.; Bagger A.; Westhead O.; Kondo Y.; Khobnya A.; Winiwarter A.; Davies B. J. V.; Walsh A.; Katayama Y.; Yamada Y.; Ryan M. P.; Titirici M.-M.; Stephens I. E. L. Searching for the Rules of Electrochemical Nitrogen Fixation. ACS Catal. 2023, 13, 14513–14522. 10.1021/acscatal.3c03951. 38026818 PMC10660346

[ref26] Singh A. R.; Rohr B. A.; Statt M. J.; Schwalbe J. A.; Cargnello M.; No̷rskov J. K. Strategies toward Selective Electrochemical Ammonia Synthesis. ACS Catal. 2019, 9, 8316–8324. 10.1021/acscatal.9b02245.

[ref27] McEnaney J. M.; Singh A. R.; Schwalbe J. A.; Kibsgaard J.; Lin J. C.; Cargnello M.; Jaramillo T. F.; No̷rskov J. K. Ammonia synthesis from N_2_ and H_2_O using a lithium cycling electrification strategy at atmospheric pressure. Energy Environ. Sci. 2017, 10, 1621–1630. 10.1039/C7EE01126A.

[ref28] Chang W.; Jain A.; Rezaie F.; Manthiram K. Lithium-mediated nitrogen reduction to ammonia via the catalytic solid–electrolyte interphase. Nature Catalysis 2024, 7, 231–241. 10.1038/s41929-024-01115-6.

[ref29] Ng K. L.; Amrithraj B.; Azimi G. Nonaqueous rechargeable aluminum batteries. Joule 2022, 6, 134–170. 10.1016/j.joule.2021.12.003.

[ref30] Leung O. M.; Schoetz T.; Prodromakis T.; Ponce de Leon C. Review—Progress in Electrolytes for Rechargeable Aluminium Batteries. J. Electrochem. Soc. 2021, 168, 056509 10.1149/1945-7111/abfb36.

[ref31] Forero-Saboya J. D.; Tchitchekova D. S.; Johansson P.; Palacín M. R.; Ponrouch A. Interfaces and Interphases in Ca and Mg Batteries. Advanced Materials Interfaces 2022, 9, 2101578 10.1002/admi.202101578.

[ref32] Forero-Saboya J.; Davoisne C.; Dedryvère R.; Yousef I.; Canepa P.; Ponrouch A. Understanding the nature of the passivation layer enabling reversible calcium plating. Energy Environ. Sci. 2020, 13, 3423–3431. 10.1039/D0EE02347G.

[ref33] Wang D.; Gao X.; Chen Y.; Jin L.; Kuss C.; Bruce P. G. Plating and stripping calcium in an organic electrolyte. Nat. Mater. 2018, 17, 16–20. 10.1038/nmat5036. 29180779

[ref34] Steinberg K.; Yuan X.; Klein C. K.; Lazouski N.; Mecklenburg M.; Manthiram K.; Li Y. Imaging of nitrogen fixation at lithium solid electrolyte interphases via cryo-electron microscopy. Nature Energy 2023, 8, 138–148. 10.1038/s41560-022-01177-5.

[ref35] Jain A.; Ong S. P.; Hautier G.; Chen W.; Richards W. D.; Dacek S.; Cholia S.; Gunter D.; Skinner D.; Ceder G.; Persson K. A. Commentary: The Materials Project: A materials genome approach to accelerating materials innovation. APL Materials 2013, 1, 011002 10.1063/1.4812323.

[ref36] Spry M.; Westhead O.; Tort R.; Moss B.; Katayama Y.; Titirici M.-M.; Stephens I. E. L.; Bagger A. Water Increases the Faradaic Selectivity of Li-Mediated Nitrogen Reduction. ACS Energy Letters 2023, 8, 1230–1235. 10.1021/acsenergylett.2c02792. 36816776 PMC9926485

[ref37] Li K.; Andersen S. Z.; Statt M. J.; Saccoccio M.; Bukas V. J.; Krempl K.; Sažinas R.; Pedersen J. B.; Shadravan V.; Zhou Y.; Chakraborty D.; Kibsgaard J.; Vesborg P. C. K.; No̷rskov J. K.; Chorkendorff I. Enhancement of lithium-mediated ammonia synthesis by addition of oxygen. Science 2021, 374, 1593–1597. 10.1126/science.abl4300. 34941415

[ref38] Westhead O.; Spry M.; Bagger A.; Shen Z.; Yadegari H.; Favero S.; Tort R.; Titirici M.; Ryan M. P.; Jervis R.; Katayama Y.; Aguadero A.; Regoutz A.; Grimaud A.; Stephens I. E. L. The role of ion solvation in lithium mediated nitrogen reduction. J. Mater. Chem. A 2023, 11, 12746–12758. 10.1039/D2TA07686A. PMC10281334 37346742

[ref39] McShane E. J.; Niemann V. A.; Benedek P.; Fu X.; Nielander A. C.; Chorkendorff I.; Jaramillo T. F.; Cargnello M. Quantifying Influence of the Solid-Electrolyte Interphase in Ammonia Electrosynthesis. ACS Energy Letters 2023, 8, 4024–4032. 10.1021/acsenergylett.3c01534.

[ref40] Li S.; Zhou Y.; Li K.; Saccoccio M.; Sažinas R.; Andersen S. Z.; Pedersen J. B.; Fu X.; Shadravan V.; Chakraborty D.; Kibsgaard J.; Vesborg P. C.; No̷rskov J. K.; Chorkendorff I. Electrosynthesis of ammonia with high selectivity and high rates via engineering of the solid-electrolyte interphase. Joule 2022, 6, 2083–2101. 10.1016/j.joule.2022.07.009. 36188748 PMC9511958

[ref41] Kani N. C.; Goyal I.; Gauthier J. A.; Shields W.; Shields M.; Singh M. R. Pathway toward Scalable Energy-Efficient Li-Mediated Ammonia Synthesis. ACS Appl. Mater. Interfaces 2024, 16, 16203–16212. 10.1021/acsami.3c19499. 38506506

[ref42] Christensen O.; Bagger A.; Rossmeisl J. The Missing Link for Electrochemical CO_2_ Reduction: Classification of CO vs HCOOH Selectivity via PCA, Reaction Pathways, and Coverage Analysis. ACS Catal. 2024, 14, 2151–2161. 10.1021/acscatal.3c04851.

[ref43] Sui Y.; Ji X. Electrolyte Interphases in Aqueous Batteries. Angew. Chem., Int. Ed. 2024, 63, e202312585 10.1002/anie.202312585. 37749061

