# Supplementary material for: Electrochemical Nitrogen Reduction: The Energetic Distance to Lithium
Source: ACS Energy Lett. 2024 Sep 19;9(10):4947–52. doi: 10.1021/acsenergylett.4c01638 (PMC11474955; doi:10.1021/acsenergylett.4c01638)
Supplement: Supplementary file 1 — nz4c01638_si_001.pdf [file nz4c01638_si_001.pdf]

Supporting information: Electrochemical Nitrogen Reduction: The Energetic Distance to Lithium

Alexander Bagger,[*,†] Romain Tort,[‡] Maria-Magdalena Titirici,[‡] Aron Walsh,[¶] and Ifan E. L. Stephens[¶]

†*Department of Physics, Technical University of Denmark, Kongens Lyngby 2800, Denmark*

‡*Department of Chemical Engineering, Imperial College London, SW7 2AZ London, United Kingdom*

¶*Department of Materials, Imperial College London, London SW7 2AZ, United Kingdom*

E-mail: alexbag@dtu.dk

Table of Content

- o Computational Details
- o Calculations of features
- o Convergence checks
- o Data table & filling missing data
- o Additional plots

Computational details

We carry out two sets of simulations for bulk materials and binding energies. All our data is presented in Table S1-S3 and accessible databases and analysis scripts are available on `https://github.com/AlexanderBagger/Energetic_distance_to_lithium`.

For bulk materials we download the most stable phases of interest from the Materials Project homepage.[1] We initiate these bulk structures with spin and optimize them to a force below 0.05 eV/Å using the RPBE functional,[2] a 800 eV planewave cutoff and an automated k-point density set to 25/cell sizes using ASE[3] and GPAW.[4,5]

For the binding energies we optimize bulk hpc, fcc and bcc structures and following build 2x2x5 structures with the three two layers fixed including initial spin. The system is then relaxed with a 500 cutoff plane wave basis set and the RPBE functional using ASE and GPAW. Convergence checks of nitrogen binding is presented in the section Convergence checks. Here it can be noticed that the relevant group 1 materials nitrogen binding energy depends strongly on the optimized lattice parameter in our setup.

For each feature calculated features, as explained in the section below, we display the feature value as a function of the standard reduction potential in Figure S6. Additionally, we co-plot a histogram and probability density function using numpy and scipy.stats packages

in python. The histogram uses the default 10 bins, and the probability density function is scales by the feature standard deviation and shifted by the mean value.

Finally, Linear regression is used to fill in missing data, and correlation and PCA analysis were carried out using the pandas[6] and sklearn[7] python packages.

Calculated features

Below we show how we calculate all features used in the analysis.

Formation energies:

$$\Delta \mathrm{H}_{Nitride} = \left(\mathrm{E}_{M_xN_y} - x\mathrm{E}_M - \tfrac{1}{2}y\mathrm{E}_{N_2}\right)/\mathrm{N}^{Atoms}_{M_xN_y} \tag{1}$$

$$\Delta \mathrm{H}_{Hydride} = \left(\mathrm{E}_{M_xH_y} - x\mathrm{E}_M - \tfrac{1}{2}y\mathrm{E}_{H_2}\right)/\mathrm{N}^{Atoms}_{M_xH_y} \tag{2}$$

$$\Delta \mathrm{H}_{Oxide} = \left(\mathrm{E}_{M_xO_y} - x\mathrm{E}_M - y(\mathrm{E}_{H_2O} - \mathrm{E}_{H_2})\right)/\mathrm{N}^{Atoms}_{M_xO_y} \tag{3}$$

$$\Delta \mathrm{H}_{M_xO_yH_z} = \left(\mathrm{E}_{M_xO_yH_z} - x\mathrm{E}_M - y(\mathrm{E}_{H_2O} - \mathrm{E}_{H_2}) - \tfrac{1}{2}z\mathrm{E}_{H_2}\right)/\mathrm{N}^{Atoms}_{M_xO_yH_z} \tag{4}$$

$$\Delta \mathrm{H}_{M_xC_yO_z} = \left(\mathrm{E}_{M_xC_yO_z} - x\mathrm{E}_M - y(\mathrm{E}_{CO} - (\mathrm{E}_{H_2O} - \mathrm{E}_{H_2})) - z(\mathrm{E}_{H_2O} - \mathrm{E}_{H_2})\right)/\mathrm{N}^{Atoms}_{M_xC_yO_z} \tag{5}$$

$$\Delta \mathrm{H}_{M_xF_y} = \left(\mathrm{E}_{M_xF_y} - x\mathrm{E}_M - y(\mathrm{E}_{HF} - \tfrac{1}{2}\mathrm{E}_{H_2})\right)/\mathrm{N}^{Atoms}_{M_xF_y} \tag{6}$$

$$\Delta \mathrm{H}_{MN_3} = \mathrm{E}_{M_3N_9} - 3\mathrm{E}_M - \tfrac{9}{2}y\mathrm{E}_{N_2} \tag{7}$$

$$\Delta \mathrm{H}_{M_3N} = \mathrm{E}_{M_3N} - 3\mathrm{E}_M - \tfrac{1}{2}y\mathrm{E}_{N_2} \tag{8}$$

$$\tag{9}$$

Phase reaction energies:

Reactions:

$$\mathrm{M}_p\mathrm{N}_q + \frac{3q}{2}\mathrm{H}_2 \rightarrow p/x\mathrm{M}_x + q\mathrm{NH}_3 \tag{10}$$

$$\mathrm{M}_p\mathrm{N}_q + \frac{3q+yp/x}{2}\mathrm{H}_2 \rightarrow p/x\mathrm{M}_x\mathrm{H}_y + q\mathrm{NH}_3 \tag{11}$$

$$\mathrm{M}_p\mathrm{N}_q + yp/x\mathrm{H}_2\mathrm{O} + \frac{3q-2yp/x}{2}\mathrm{H}_2 \rightarrow p/x\mathrm{M}_x\mathrm{O}_y + q\mathrm{NH}_3 \tag{12}$$

$$\mathrm{M}_p\mathrm{N}_q + \mathrm{M}_x + \mathrm{H}_2\mathrm{O} + \frac{3q}{2}\mathrm{H}_2 \rightarrow p/x\mathrm{M}_x\mathrm{O}_y\mathrm{H}_z + q\mathrm{NH}_3 \tag{13}$$

$$\tag{14}$$

Energetic calculation:

$$\Delta\mathrm{E}_{M_x} = p/x\mathrm{E}_{M_x} - \mathrm{E}_{M_pN_q} + q\mathrm{E}_{NH_3} - \frac{3q}{2}\mathrm{E}_{H_2} \tag{15}$$

$$\Delta\mathrm{E}_{M_xH_y} = p/x\mathrm{E}_{M_xH_y} - \mathrm{E}_{M_pN_q} + q\mathrm{E}_{NH_3} - \frac{3q+yp/x}{2}\mathrm{E}_{H_2} \tag{16}$$

$$\Delta\mathrm{E}_{M_xO_y} = p/x\mathrm{E}_{M_xO_y} - \mathrm{E}_{M_pN_q} + q\mathrm{E}_{NH_3} - \frac{3q}{2}\mathrm{E}_{H_2} - yp/x\left(\mathrm{E}_{H_2O} - \frac{1}{2}\mathrm{E}_{H_2}\right) \tag{17}$$

$$\Delta\mathrm{E}_{M_xO_yH_z} = p/x\mathrm{E}_{M_xO_yH_z} - \mathrm{E}_{M_pN_q} + q\mathrm{E}_{NH_3} - \frac{3q+zp/x}{2}\mathrm{E}_{H_2} - yp/x\left(\mathrm{E}_{H_2O} - \frac{1}{2}\mathrm{E}_{H_2}\right) \tag{18}$$

$$\tag{19}$$

Binding energies:

$$\Delta\mathrm{E}_{\ {*N_2}} = \mathrm{E}_{\ {*N_2}} - \mathrm{E}_{\ *} - \mathrm{E}_{N_2} \tag{20}$$

$$\Delta\mathrm{E}_{\ {*N}} = \mathrm{E}_{\ {*N}} - \mathrm{E}_{\ *} - \frac{1}{2}\mathrm{E}_{N_2} \tag{21}$$

$$\Delta\mathrm{E}_{\ {*NH_2}} = \mathrm{E}_{\ {*NH_2}} - \mathrm{E}_{\ *} - \frac{1}{2}\mathrm{E}_{N_2} - \mathrm{E}_{H_2} \tag{22}$$

$$\Delta\mathrm{E}_{\ {*NH_3}} = \mathrm{E}_{\ {*NH_3}} - \mathrm{E}_{\ *} - \frac{1}{2}\mathrm{E}_{N_2} - \frac{3}{2}\mathrm{E}_{H_2} \tag{23}$$

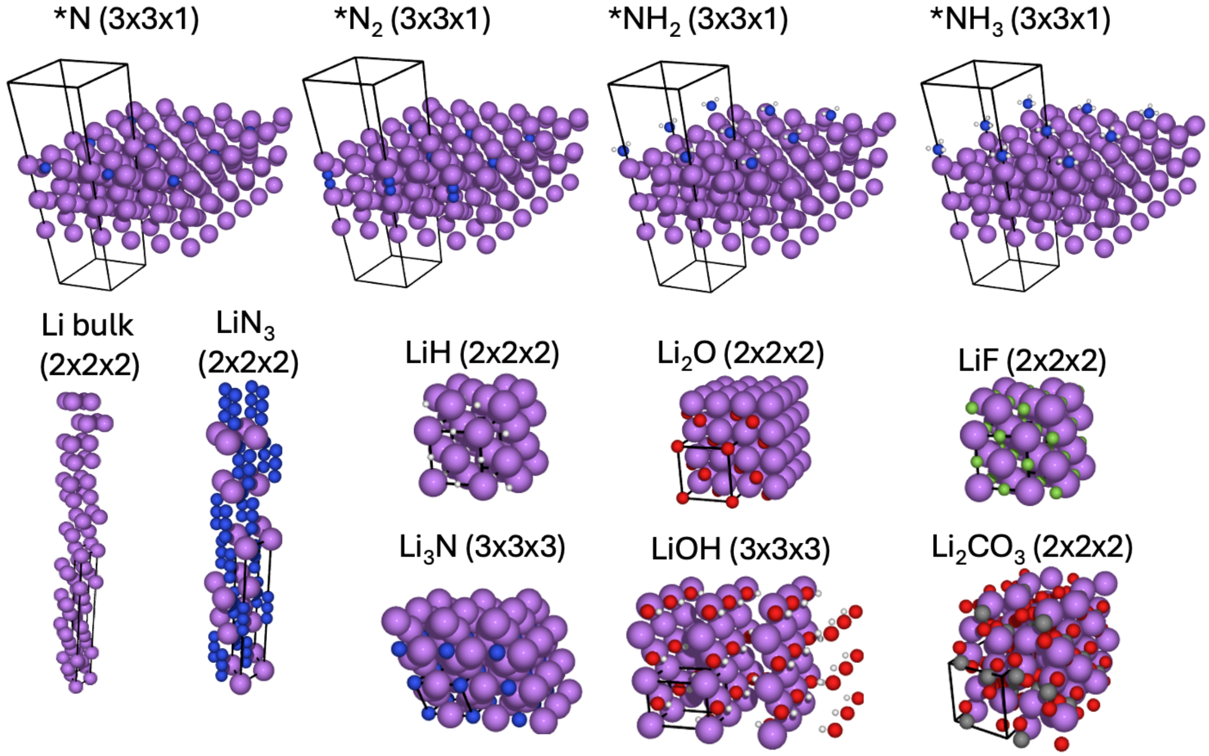

Figure S1: Calculated Lithium structures with the original cell drawn and for each noted how many times the cell is repeated for visualisation.

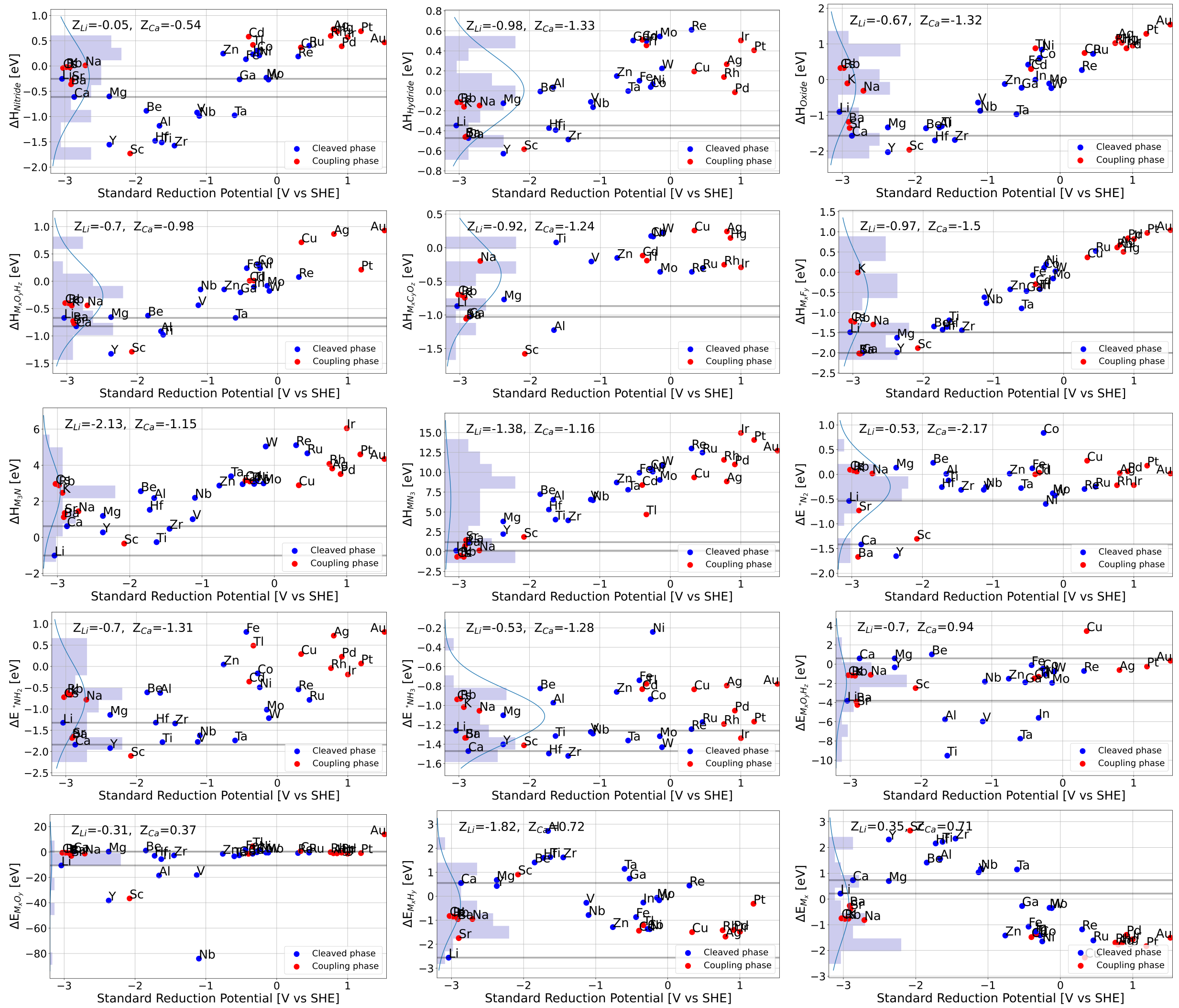

Figure S2: Calculated features versus the standard reduction potential. Horizontal lines indicate Lithium and Calcium (working materials), a histogram and a probability density distribution is plotted together with the standard score (Z) values for Lithium and Calcium.

Convergence checks

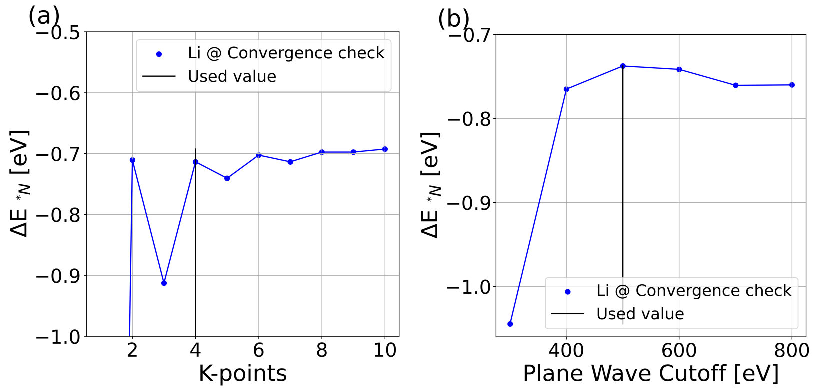

Figure S3: Convergence checks of nitrogen binding energy (ΔE_{*N}) on Lithium as a function of (a) K-points and (b) Plane wave cutoff. Used value is the vertical black line.

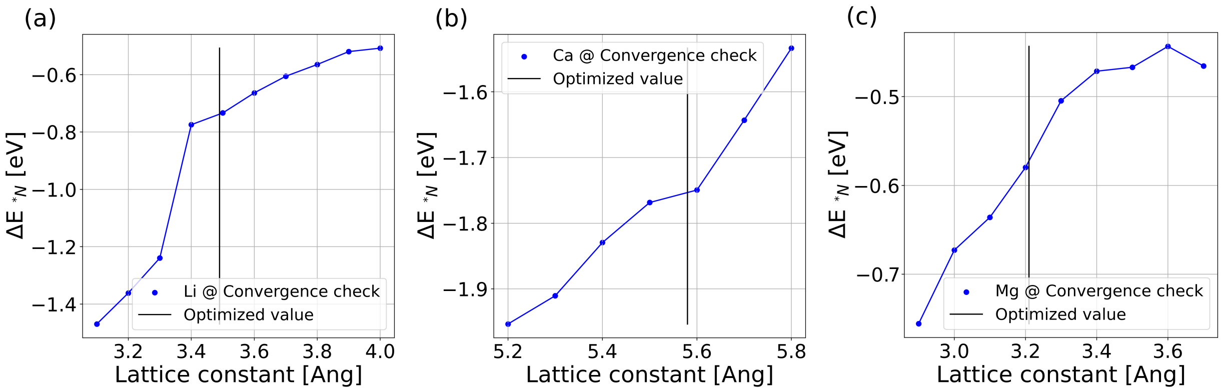

Figure S4: Convergence checks of nitrogen binding energy (ΔE_{*N}) as a function of lattice constant on (a) Lithium, (b) Calcium and (c) Magnesium. Lattice parameter optimized and used value are the vertical black line. Note the strong binding of nitrogen when having a too small lattice parameter.

Data table & filling missing data

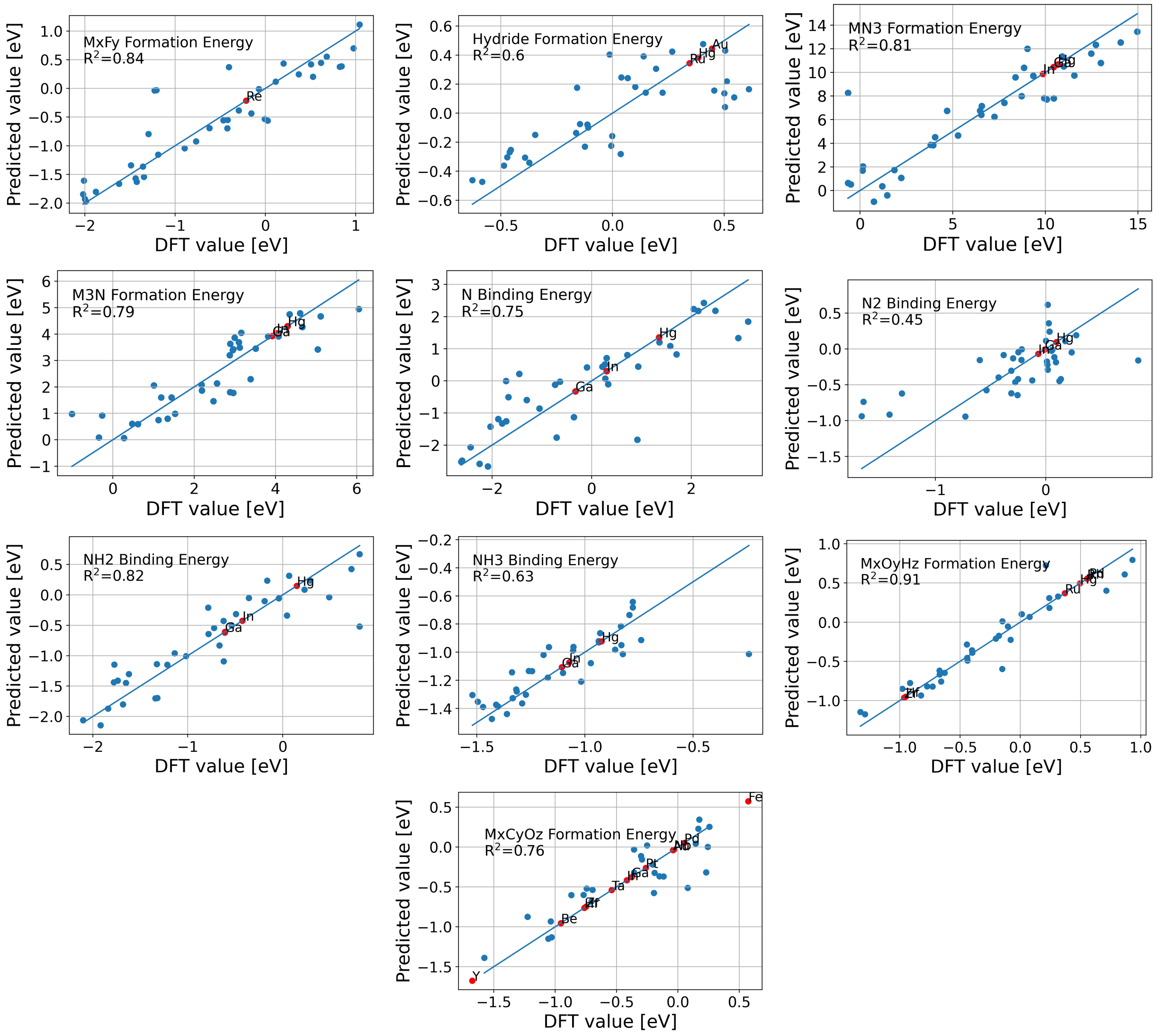

Figure S5: Using a linear regression model we predict missing values (noted by red dot and text). We first predict variables with the least missing points (ie. MxFy Formation Energy missing only 'Re'), following we include this now full feature to predict the next (Hydride Formation Energy) until using 13 features to predict MxCyOz Formation energy

Table S1: Table of formation energies (continues below). Diss=True means that the material forms a nitride phase with isolated nitrogen atoms. Black numbers are calculated by DFT and red numbers are predicted from linear regression (see Figure S5). All values are in eV

**Name**	**Diss**	V_{SHE}	$\Delta H_{Nitride}$	$\Delta H_{Hydride}$	ΔH_{Oxide}	$\Delta H_{M_xO_yH_z}$	$\Delta H_{M_xC_yO_z}$	$\Delta H_{M_xF_y}$
**Co**	True	-0.28	0.31	0.04	0.61	0.31	0.17	0.11
**Hg**	False	0.85	0.68	0.38	1.04	0.5	0.15	0.51
**Tl**	False	-0.34	0.42	0.46	0.88	0.01	-0.19	-0.4
**Pt**	False	1.19	0.69	0.41	1.28	0.21	-0.26	0.98
**Rb**	False	-2.98	-0.02	-0.11	0.33	-0.4	-0.7	-1.23
**Mg**	True	-2.37	-0.6	-0.12	-1.34	-0.66	-0.77	-1.62
**Cs**	False	-3.03	-0.04	-0.11	0.33	-0.4	-0.7	-1.21
**Y**	True	-2.37	-1.55	-0.63	-2.03	-1.33	-1.68	-1.99
**In**	True	-0.34	0.23	0.5	-0.0	-0.1	-0.42	-0.42
**V**	True	-1.13	-0.92	-0.11	-0.64	-0.44	-0.2	-0.62
**Zr**	True	-1.45	-1.57	-0.49	-1.69	-0.96	-0.75	-1.44
**Al**	True	-1.66	-1.18	0.04	-1.34	-0.91	-1.23	-1.36
**Ru**	True	0.46	0.4	0.34	0.73	0.37	-0.3	0.53
**Ti**	True	-1.63	-1.51	-0.39	-1.3	-0.98	0.08	-1.19
**Ir**	False	1.0	0.57	0.5	0.95	0.55	-0.29	0.82
**Sc**	False	-2.08	-1.73	-0.58	-1.96	-1.29	-1.58	-1.88
**K**	False	-2.93	-0.03	-0.16	-0.1	-0.44	-0.74	-0.01
**Ag**	False	0.8	0.73	0.27	1.18	0.87	0.24	0.68
**Au**	False	1.52	0.47	0.44	1.54	0.93	-0.04	1.04
**Ta**	True	-0.6	-0.97	-0.0	-0.97	-0.67	-0.54	-0.9
**Mo**	True	-0.15	-0.23	0.54	-0.11	-0.08	-0.36	-0.16
**Ni**	True	-0.25	0.21	0.07	0.84	0.24	0.17	0.2
**Ca**	True	-2.87	-0.61	-0.47	-1.57	-0.82	-1.03	-2.0
**Fe**	True	-0.44	0.14	0.1	0.42	0.24	0.57	-0.07
**W**	True	-0.12	-0.27	0.22	-0.24	-0.18	0.23	0.02
**Nb**	True	-1.1	-0.99	-0.16	-0.87	-0.15	-0.03	-0.77
**Ba**	False	-2.91	-0.36	-0.46	-1.18	-0.73	-1.06	-2.01
**Sr**	False	-2.9	-0.28	-0.46	-1.35	-0.77	-1.04	-2.02
**Rh**	False	0.76	0.6	0.14	1.02	0.56	-0.25	0.61
**Hf**	True	-1.72	-1.48	-0.37	-1.7	-0.95	-0.76	-1.43
**Re**	True	0.3	0.19	0.61	0.27	0.08	-0.36	-0.21
**Cd**	False	-0.4	0.58	0.51	0.3	0.01	-0.12	-0.3
**Ga**	True	-0.53	-0.26	0.5	-0.23	-0.2	-0.38	-0.47
**Pd**	False	0.92	0.39	-0.01	0.88	0.57	0.05	0.84
**Be**	True	-1.85	-0.89	-0.01	-1.36	-0.63	-0.95	-1.35
**Zn**	True	-0.76	0.25	0.15	-0.12	-0.15	-0.15	-0.42
**Na**	False	-2.71	0.01	-0.15	-0.31	-0.44	-0.2	-1.3
**Li**	True	-3.04	-0.25	-0.35	-0.9	-0.67	-0.87	-1.49
**Cu**	False	0.34	0.37	0.19	0.75	0.71	0.26	0.37

Table S2: Table of formation and binding energies (continues below). Diss=True means that the material forms a nitride phase with isolated nitrogen atoms. Black numbers are calculated by DFT and red numbers are predicted from linear regression (see Figure S5). All values are in eV.

**Name**	**Diss**	$\mathbf{V}_{SHE}$	$\Delta\mathbf{H}_{MN_3}$	$\Delta\mathbf{H}_{M_3N}$	$\Delta\mathbf{E}_{*N}$	$\Delta\mathbf{E}_{*N_2}$	$\Delta\mathbf{E}_{*NH_2}$	$\Delta\mathbf{E}_{*NH_3}$
**Co**	True	-0.28	10.45	2.96	0.27	0.84	-0.16	-0.94
**Hg**	False	0.85	10.66	4.3	1.35	0.1	0.15	-0.92
**Tl**	False	-0.34	4.7	3.1	2.25	0.03	0.49	-0.78
**Pt**	False	1.19	14.07	4.6	0.72	0.18	0.07	-1.17
**Rb**	False	-2.98	-0.5	2.88	2.48	0.08	-0.62	-0.93
**Mg**	True	-2.37	3.81	1.19	-0.36	0.14	-1.14	-1.1
**Cs**	False	-3.03	-0.65	2.96	2.05	0.09	-0.72	-0.94
**Y**	True	-2.37	2.22	0.28	-2.08	-1.65	-1.92	-1.4
**In**	True	-0.34	9.87	4.03	0.3	-0.07	-0.42	-1.07
**V**	True	-1.13	6.57	1.01	-1.88	-0.31	-1.77	-1.27
**Zr**	True	-1.45	3.95	0.47	-2.6	-0.31	-1.34	-1.52
**Al**	True	-1.66	6.55	2.18	-0.71	0.01	-0.62	-0.97
**Ru**	True	0.46	12.48	4.66	0.21	-0.25	-0.79	-1.17
**Ti**	True	-1.63	4.05	-0.26	-2.43	-0.12	-1.78	-1.32
**Ir**	False	1.0	14.96	6.05	0.27	-0.22	-0.19	-1.34
**Sc**	False	-2.08	1.85	-0.34	-2.25	-1.3	-2.1	-1.41
**K**	False	-2.93	-0.65	2.47	2.14	0.05	-0.67	-1.02
**Ag**	False	0.8	8.85	3.82	3.14	0.03	0.72	-0.79
**Au**	False	1.52	12.73	4.35	2.94	0.02	0.81	-0.78
**Ta**	True	-0.6	7.79	3.39	-2.03	-0.28	-1.74	-1.36
**Mo**	True	-0.15	9.04	2.99	-1.45	-0.38	-1.02	-1.32
**Ni**	True	-0.25	10.07	3.12	-0.1	-0.6	-0.49	-0.24
**Ca**	True	-2.87	1.19	0.62	-1.72	-1.42	-1.84	-1.47
**Fe**	True	-0.44	9.94	2.95	-1.72	0.12	0.81	-0.74
**W**	True	-0.12	10.91	5.04	-1.67	-0.43	-1.22	-1.43
**Nb**	True	-1.1	6.47	2.19	-1.8	-0.25	-1.62	-1.29
**Ba**	False	-2.91	0.75	1.12	-1.31	-1.67	-1.68	-1.33
**Sr**	False	-2.9	1.46	1.35	-1.05	-0.73	-1.65	-1.33
**Rh**	False	0.76	11.56	4.07	0.3	-0.22	-0.04	-1.19
**Hf**	True	-1.72	5.29	1.53	-2.62	-0.26	-1.32	-1.5
**Re**	True	0.3	13.0	5.11	-0.63	-0.3	-0.54	-1.24
**Cd**	False	-0.4	8.39	3.16	1.58	0.0	-0.36	-0.83
**Ga**	True	-0.53	10.45	3.92	-0.33	-0.01	-0.61	-1.11
**Pd**	False	0.92	10.99	3.51	0.93	0.06	0.23	-1.05
**Be**	True	-1.85	7.25	2.56	0.92	0.23	-0.61	-0.82
**Zn**	True	-0.76	8.72	2.87	0.33	0.02	0.05	-0.86
**Na**	False	-2.71	0.16	1.44	1.36	0.02	-0.78	-1.06
**Li**	True	-3.04	0.14	-1.01	-0.74	-0.54	-1.33	-1.26
**Cu**	False	0.34	9.35	2.89	1.7	0.28	0.29	-0.83

Table S3: Table of phase reaction energies (Diss=True means that the material forms a nitride phase with isolated nitrogen atoms). All values are in eV. For phase reaction energies missing data/empty space is not filled by linear regression.

Name	Diss	V_{SHE}	$\Delta E_{M_xO_yH_z}$	$\Delta E_{M_xO_y}$	$\Delta E_{M_xH_y}$	ΔE_{M_x}
**Co**	True	-0.28	-0.47	-0.19	-1.36	-1.41
**Hg**	False	0.85		-1.02		-1.71
**Tl**	False	-0.34	-1.31	4.5	-1.21	-1.36
**Pt**	False	1.19	-0.25	-0.87	-0.31	-1.83
**Rb**	False	-2.98	-1.17	-0.55	-0.85	-0.77
**Mg**	True	-2.37	0.6	0.36	0.68	0.7
**Cs**	False	-3.03	-1.15	-0.53	-0.82	-0.75
**Y**	True	-2.37	-0.34	-38.25	0.43	2.31
**In**	True	-0.34	-5.59	-1.33	-0.26	-1.26
**V**	True	-1.13	-5.96	-18.17	-0.27	1.04
**Zr**	True	-1.45		-2.72	1.62	2.35
**Al**	True	-1.66	-5.75	-18.49	2.72	1.56
**Ru**	True	0.46		-0.52		-1.61
**Ti**	True	-1.63	-9.52	-5.6	1.63	2.22
**Ir**	False	1.0		-1.32	-1.48	-1.56
**Sc**	False	-2.08	-2.5	-36.57	0.91	2.66
**K**	False	-2.93	-1.21	-0.87	-0.87	-0.76
**Ag**	False	0.8	-0.62	-0.99	-1.68	-1.77
**Au**	False	1.52	0.36	13.93		-1.5
**Ta**	True	-0.6	-7.76	-3.36	1.14	1.15
**Mo**	True	-0.15	-1.95	-0.42	-0.07	-0.34
**Ni**	True	-0.25	-1.05	1.72	-1.37	-1.65
**Ca**	True	-2.87	0.6	0.34	0.55	0.73
**Fe**	True	-0.44	-0.11	2.09	-0.87	-1.07
**W**	True	-0.12	-0.77	-0.51	-0.17	-0.35
**Nb**	True	-1.1	-1.82	-84.01	-0.78	1.17
**Ba**	False	-2.91	-3.9	-1.44	-0.95	-0.26
**Sr**	False	-2.9	-4.24	-3.08	-1.75	-0.38
**Rh**	False	0.76		-0.16	-1.42	-1.69
**Hf**	True	-1.72		-2.94	1.6	2.16
**Re**	True	0.3	-0.71	-0.82	0.45	-1.18
**Cd**	False	-0.4	-1.48	-1.43	-1.44	-1.48
**Ga**	True	-0.53	-1.89	-2.55	0.73	-0.27
**Pd**	False	0.92		-0.51	-1.4	-1.39
**Be**	True	-1.85	1.02	1.25	1.41	1.42
**Zn**	True	-0.76	-1.51	-1.43	-1.29	-1.42
**Na**	False	-2.71	-1.11	-1.23	-0.95	-0.82
**Li**	True	-3.04	-3.81	-10.6	-2.56	0.21
**Cu**	False	0.34	3.44	0.72	-1.49	-2.27

Additional plots

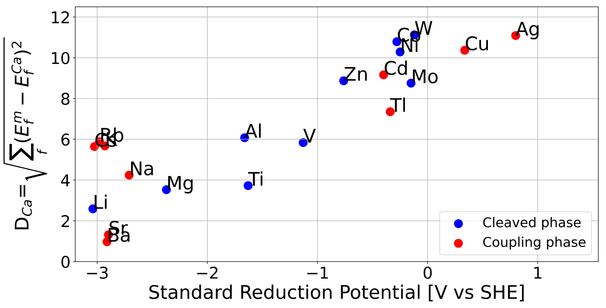

Figure S6: The distance calculated to Calcium using the formation energies and binding energies as a function of the standard reduction potential. Interestingly, it shows that Sr, Ba and Li is the materials closest to Calcium.

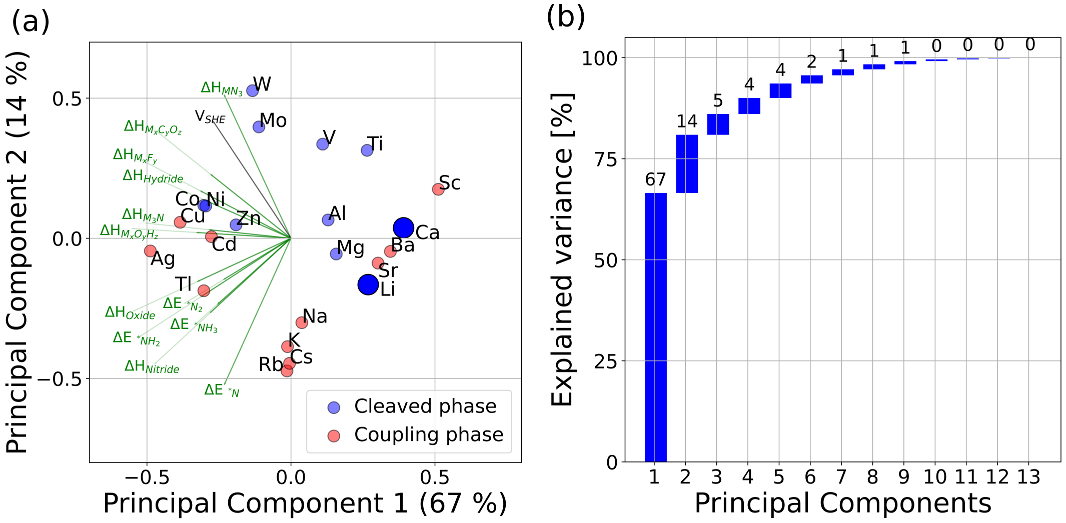

Figure S7: Principal component analysis of the nitrogen reduction feature space: formation energies (ΔH), binding energies (ΔE) and the standard reduction potential (V_{SHE}) for (a) DFT energies and (b) depicting all 13 principal components and their explained variance ratio. Data is shown in Table S1-S2.

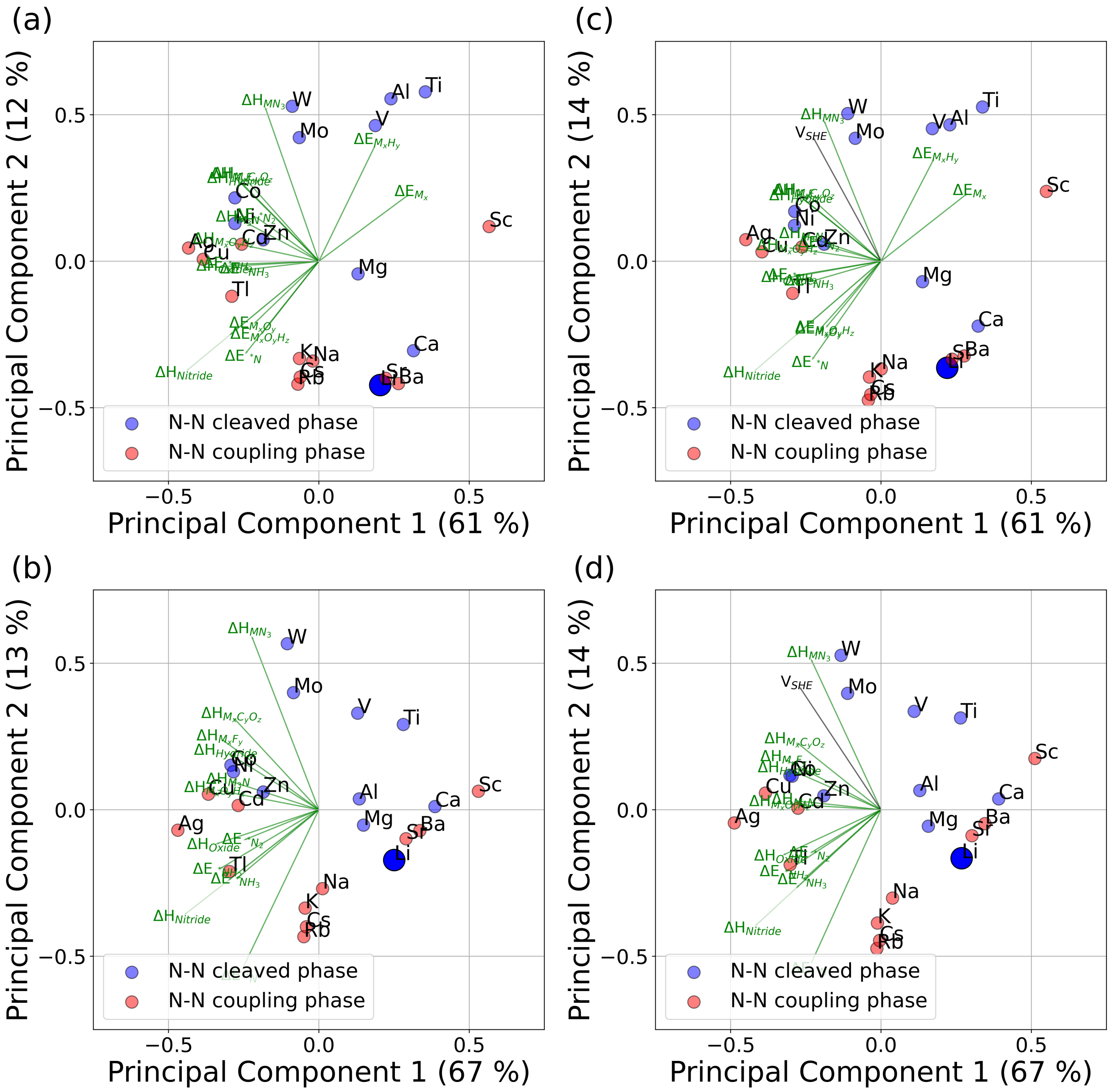

Figure S8: Principal component analysis of (a) the features (b) the features included the standard reduction potential (V_{SHE}), (c) the formation energies and binding energy features, and (d) the formation-, binding energy features and the standard reduction potential (V_{SHE}). The PCA plots without the V_{SHE} captures the close materials independent on potential, while including the potential shows almost no changes.

References

(1) Jain, A.; Ong, S. P.; Hautier, G.; Chen, W.; Richards, W. D.; Dacek, S.; Cholia, S.; Gunter, D.; Skinner, D.; Ceder, G.; Persson, K. A. Commentary: The Materials Project: A materials genome approach to accelerating materials innovation. *APL Materials* **2013**, *1*, 011002.

(2) Hammer, B.; Hansen, L. B.; Nørskov, J. K. Improved adsorption energetics within density-functional theory using revised Perdew-Burke-Ernzerhof functionals. *Phys. Rev. B* **1999**, *59*, 7413–7421.

(3) Larsen, A. H.; Mortensen, J. J.; Blomqvist, J.; Castelli, I. E.; Christensen, R.; Dułak, M.; Friis, J.; Groves, M. N.; Hammer, B.; Hargus, C.; Hermes, E. D.; Jennings, P. C.; Jensen, P. B.; Kermode, J.; Kitchin, J. R.; Kolsbjerg, E. L.; Kubal, J.; Kaasbjerg, K.; Lysgaard, S.; Maronsson, J. B.; Maxson, T.; Olsen, T.; Pastewka, L.; Peterson, A.; Rostgaard, C.; Schiøtz, J.; Schütt, O.; Strange, M.; Thygesen, K. S.; Vegge, T.; Vilhelmsen, L.; Walter, M.; Zeng, Z.; Jacobsen, K. W. The atomic simulation environment—a Python library for working with atoms. *Journal of Physics: Condensed Matter* **2017**, *29*, 273002.

(4) Mortensen, J. J.; Hansen, L. B.; Jacobsen, K. W. Real-space grid implementation of the projector augmented wave method. *Phys. Rev. B* **2005**, *71*, 035109.

(5) Enkovaara, J.; Rostgaard, C.; Mortensen, J. J.; Chen, J.; Du|ak, M.; Ferrighi, L.; Gavnholt, J.; Glinsvad, C.; Haikola, V.; Hansen, H. A.; Kristoffersen, H. H.; Kuisma, M.; Larsen, A. H.; Lehtovaara, L.; Ljungberg, M.; Lopez-Acevedo, O.; Moses, P. G.; Ojanen, J.; Olsen, T.; Petzold, V.; Romero, N. A.; Stausholm-Møller, J.; Strange, M.; Tritsaris, G. A.; Vanin, M.; Walter, M.; Hammer, B.; Häkkinen, H.; Madsen, G. K. H.; Nieminen, R. M.; Nørskov, J. K.; Puska, M.; Rantala, T. T.; Schiøtz, J.; Thygesen, K. S.; Jacobsen, K. W. Electronic structure calculations with GPAW: a real-space implemen-

tation of the projector augmented-wave method. *Journal of Physics: Condensed Matter* **2010**, *22*, 253202.

(6) McKinney, W. Data Structures for Statistical Computing in Python. Proceedings of the 9th Python in Science Conference. 2010; pp 51 – 56.

(7) Pedregosa, F.; Varoquaux, G.; Gramfort, A.; Michel, V.; Thirion, B.; Grisel, O.; Blondel, M.; Prettenhofer, P.; Weiss, R.; Dubourg, V.; Vanderplas, J.; Passos, A.; Cournapeau, D.; Brucher, M.; Perrot, M.; Duchesnay, E. Scikit-learn: Machine Learning in Python. *Journal of Machine Learning Research* **2011**, *12*, 2825–2830.
